# First case report of erlotinib plus ramucirumab treatment for lung carcinosarcoma with EGFR L858R mutation

**DOI:** 10.1111/1759-7714.15134

**Published:** 2023-10-14

**Authors:** Naoya Ishibashi, Toshiharu Tabata, Ryo Nonomura, Yutaka Oshima, Takanobu Sasaki, Hideki Mitomo, Takafumi Sugawara, Motoyasu Sagawa

**Affiliations:** ^1^ Department of Thoracic Surgery Tohoku Medical and Pharmaceutical University Hospital Sendai City Japan

**Keywords:** EGFR L858R, erlotinib, lung carcinosarcoma, non‐small cell lung cancer, ramucirumab

## Abstract

Lung carcinosarcoma is acknowledged as a rare form of lung cancer. Due to its rarity, the inability to conduct large‐scale clinical trials and interventions is currently carried out based on empirical evidence. In this study, we report the case of a 73‐year‐old female patient diagnosed with postoperative recurrence of lung carcinosarcoma. The resected tumor was diagnosed as lung carcinosarcoma, and genetic testing revealed the presence of the epidermal growth factor receptor (EGFR) exon21 L858R. Approximately 2 years postoperatively, the tumor recurred and the patient was treated with erlotinib plus ramucirumab, which were effective in controlling metastatic disease. Erlotinib plus ramucirumab is therefore a treatment option for *EGFR* mutation‐positive lung carcinosarcoma.

## Graphical abstract

This case report describes a patient with recurrent lung cancer sarcoma harboring the *EGFR* L858R mutation who was treated with a combination of an EGFR TKI and an angiogenesis inhibitor and achieved tumor shrinkage for more than 16 months. This is the first report where an EGFR TKI may be a treatment option for patients with *EGFR* mutations in lung cancer sarcomas for which no effective treatment is known.

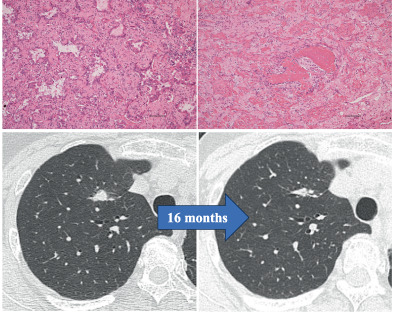

## INTRODUCTION

Lung carcinosarcoma is a rare malignant lung tumor. There are no effective drugs, and empirical pharmacotherapy is currently used in cases of inoperability or recurrence. In this report, we describe the efficacy of erlotinib plus ramucirumab for postoperative recurrent lung carcinosarcoma with epidermal growth factor receptor (EGFR) exon21 L858R positivity.

## CASE REPORT

**FIGURE 1:**
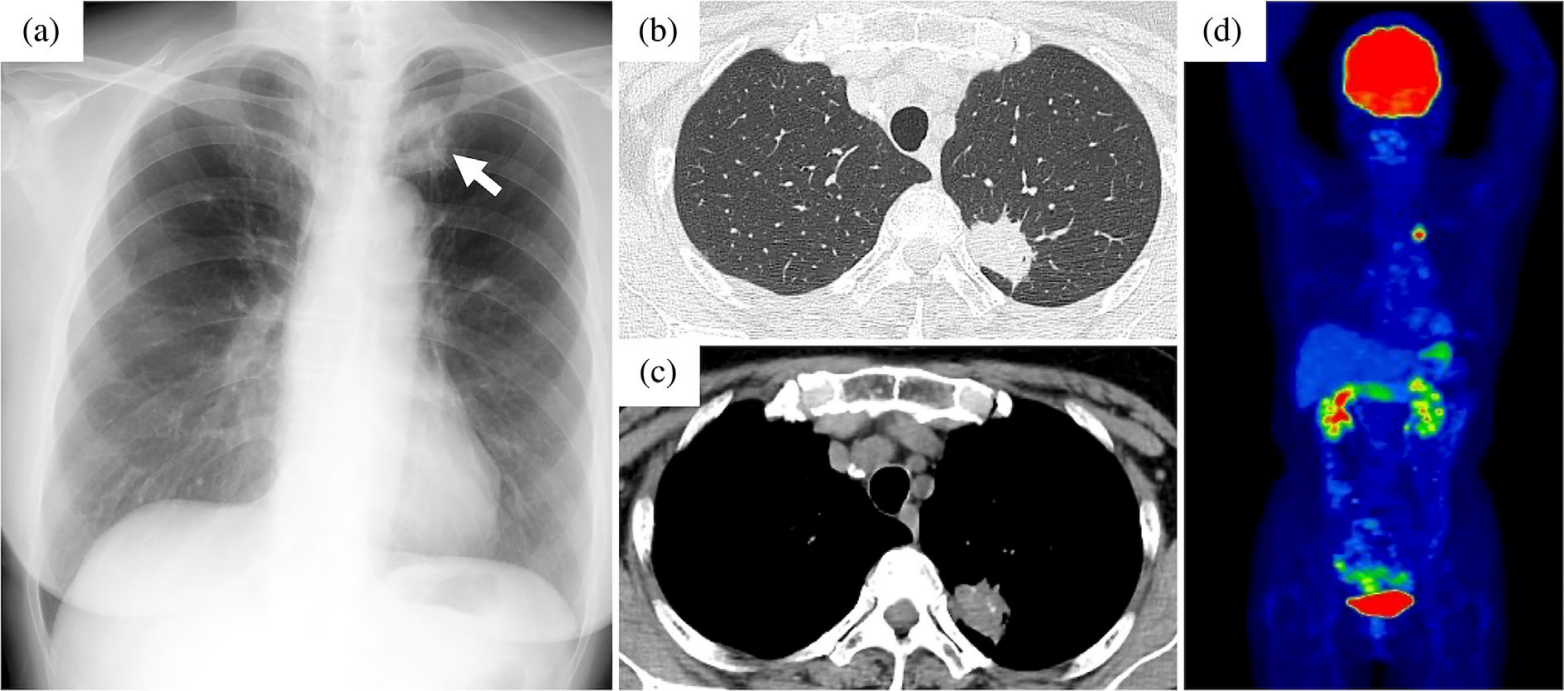
Chest x‐ray film on admission showed a tumor shadow in the left upper lung field (arrow) (a). Chest computed tomography (CT) on admission (b). CT showed a tumor with internal calcification in the left upper lobe (c). Positron emission tomography (PET) showed ^18^F‐ fluorodeoxyglucose accumulation consistent with a lung tumor (d).

**FIGURE 2:**
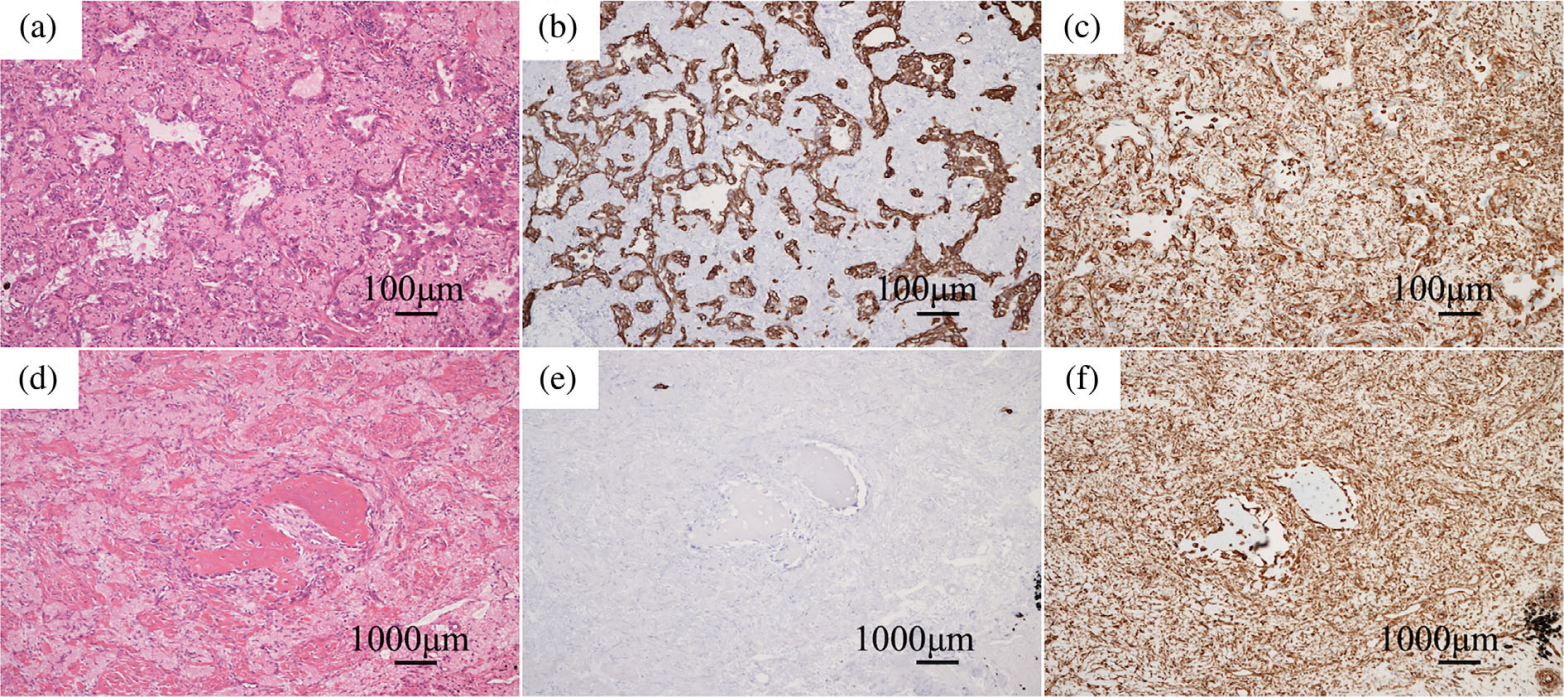
Histological features. Hematoxylin and eosin (H&E) staining showed a carcinoma component (hypodense papillary adenocarcinoma) in about 70% and a mixed sarcoma component in about 30%. The sarcoma component contained proliferating spindle‐sharped tumor cells with partial osteoid formation. Cytokeratin staining was positive for the adenocarcinoma component and negative for the sarcoma component. Vimentin staining was positive for both adenocarcinoma and sarcoma components. (a,b,c) Adenocarcinoma components, (d,e,f) sarcoma components. (a,d) H&E, (b,e) cytokeratin staining, (c,f) vimentin staining.

**FIGURE 3:**
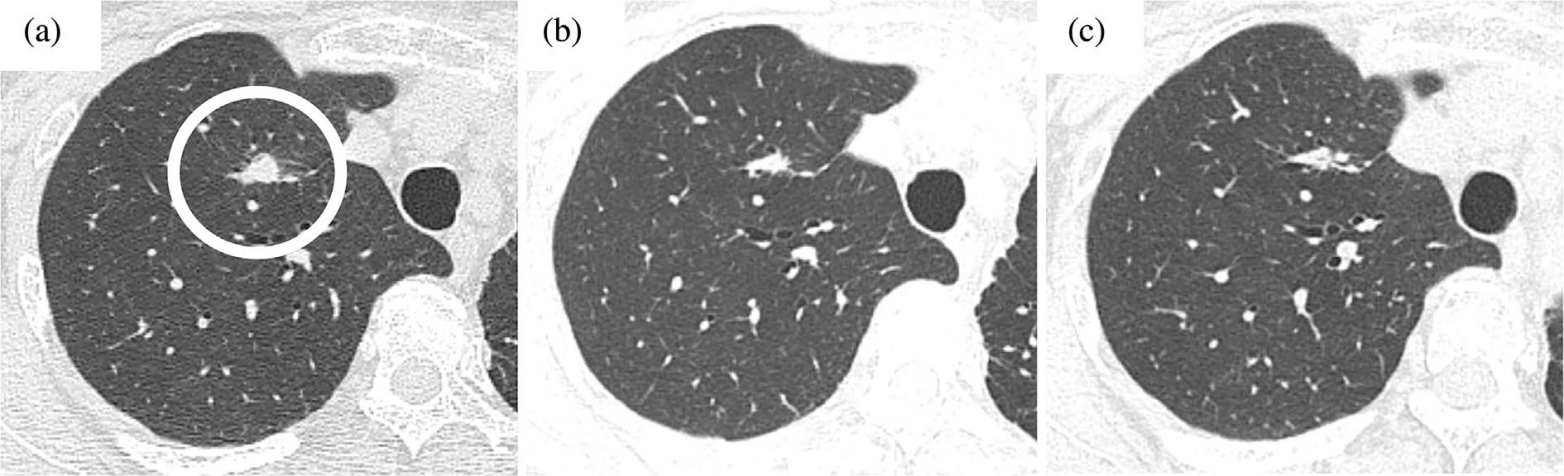
(a) Chest computed tomography (CT) showed a recurrent lesion in the right upper lobe (circle) after 2 years post‐surgery. Combination therapy with erlotinib plus ramucirumab resulted in tumor shrinkage after (b) 3 months and (c) 16 months.

A 73‐year‐old female patient was referred to our department after an abnormal shadow was found on a chest x‐ray examination during a health checkup (Figure 1a). Computed tomography (CT) of the chest revealed a 28 mm tumor with calcification in the left upper lobe (Figure 1b,c), and positron emission tomography (PET)/CT identified [^18^F] fluorodeoxyglucose uptake in the tumor (Figure 1d). The patient was clinically suspected of having left lung cancer according to the Union for International Cancer Control (UICC) T1cN0M0 stageIA3 and video‐assisted thoracoscopic surgery (VATS) left upper lobectomy and hilar and mediastinal lymph node dissection were performed. Pathological examination revealed a maximum diameter of 35 mm and papillary adenocarcinoma components in approximately 70% of the specimen and spindle‐shaped sarcoma‐like components in approximately 30%. Focal areas of osteosarcoma‐like components were observed, confirming a diagnosis of lung carcinosarcoma and the patient was finally pathologically diagnosed with T2aN0M0 stage IB (Figure 2a–f). Genetic analysis by reverse transcription‐polymerase chain reaction using tumor detected EGFR exon21 L858R mutation. The programmed death ligand 1 expression was 15% (Dako 22C3). The patient was followed up because there was no known effective adjuvant therapy for lung carcinosarcoma, and the patient did not wish to undergo adjuvant therapy. However, 2 years post‐surgery, a small nodule appeared in the right lung segment 1 on chest CT and showed a progression (Figure 3a). When we diagnosed a postoperative recurrence and presented her with a treatment plan (surgical resection or drug therapy), she chose drug therapy. The diagnosis of postoperative recurrence led to the initiation of erlotinib (150 mg/day, daily) and ramucirumab (10 mg/kg, every two weeks). Adverse events were fatigue as a common terminology adverse event grade 1, skin rash as grade 2, and oral mucositis as grade 2. Erlotinib was reduced to every other day and treatment is still ongoing. The patient's subsequent clinical course has been good, with partial response (revised RESCIST guidelines version 1.1) lasting more than 16 months (Figure 3b,c).

## DISCUSSION

Lung carcinosarcoma is a rare tumor, accounting for 0.2%–0.3% of all lung cancers. It is defined as a biphasic tumor with malignant components of both epithelial and mesenchymal origins.^1^, ^2^ Because of its biphasic nature, only one component may be obtained in the case of small biopsy specimens. Thus, preoperative diagnosis is difficult in the case of peripheral lesions, and even tumors arising centrally may be difficult to diagnose accurately.^3^, ^4^ Koss et al. reported that lung carcinosarcoma has a 5‐year survival rate of 21.3%.^5^ On the other hand, Petrov et al. analyzed 15 cases and reported that 5‐year survival rate of 49.38% postoperatively.^6^ Since there is no established effective drug therapy for lung carcinosarcoma, aggressive surgical resection should be considered in clinically operable patients. If curative surgery is not possible at the time of diagnosis, platinum‐based chemotherapy is primarily used, such as in non‐small cell lung cancer (NSCLC). There have also been reports where cisplatin and doxorubicin have been used, following the treatment regimen for soft tissue malignancies.^7^ However, due to the limited number of cases and the difficulty in conducting large‐scale clinical trials for effective treatment, the treatment strategy is left to the discretion of individual physicians.

Osimertinib has become the standard treatment for first‐line therapy in patients with *EGFR* gene mutation‐positive NSCLC.^8^ However, subgroup analysis of the FLAURA trial showed that the median progression‐free survival (PFS) in the *EGFR* exon21 L858R mutation‐positive group did not show the same marked benefit as seen in the *EGFR* exon19 deletion (del19) mutation‐positive group (L858R: 14.4 months, del19: 21.4 months).^9^ Different mutations in the genes may result in varying sensitivities to tyrosine kinase inhibitors (TKIs). On the other hand, the RELAY trial (erlotinib plus ramucirumab) and CTONG1509 trial (erlotinib plus bevacizumab) were conducted using combination therapy of a TKI and angiogenesis inhibitor, and the median PFS (del19/L858R) was comparable, 19.6/19.4 months for the RELAY trial and 17.7/19.5 months for the CTONG1509 trial, respectively.^10^, ^11^ The results based on the genetic mutations in these clinical trials cannot be easily and directly compared. However, the addition of anti‐VEGFR2 antibody to a TKI can significantly reduce the number of CD31‐positive blood vessels and potentiate the antitumor effect of the TKI, in addition to inhibiting angiogenesis. Furthermore, in another study, VEGFR2 expression was induced after TKI treatment, suggesting the importance of VEGFR2 inhibitor combination.^12^ In cases of recurrence after the use of first‐ and second‐generation TKIs, sequencing therapy with osimertinib can be an option when T790M is identified.^13^ Simulated PFS of a representative clinical trial examining the efficacy of an EGFR TKI for NSCLC showed a PFS of 24.8 months for osimertinib to chemotherapy sequencing. Although T790M is positive in about 40% of patients when a TKI is used as first‐line therapy, erlotinib plus ramucirumab treatment is predicted to have a PFS of 28.1 months, which is superior to osimertinib.^14^ Considering these reports and the fact that a Japanese patient was entered in the RELAY trial, we chose erlotinib plus ramucirumab therapy over erlotinib plus bevacizumab therapy for postoperative recurrence of lung carcinosarcoma with *EGFR* exon 21 L858R mutation. Osimertinib remained as sequence therapy after this treatment became resistant. Currently, a partial response (revised RECIST guidelines version 1.1) has been maintained for approximately 50 months, and the patient's condition is well controlled. Although there is no established effective drug therapy for lung carcinosarcoma, TKI therapy should be considered as an option in cases of *EGFR* gene mutation‐positive tumors, and erlotinib plus ramucirumab combination therapy may be particularly effective in cases with the *EGFR* ezon21 L858R mutation.

## AUTHOR CONTRIBUTIONS

Naoya Ishibashi, Toshiharu Tabata, Ryo Nonomura, Yutaka Oshima, Takanobu Sasaki, Hideki Mitomo, Takafumi Sugawara and Motoyasu Sagawa drafted the manuscript and contributed to the treatment of the patient. All authors have read and approved the final manuscript.

## CONFLICT OF INTEREST STATEMENT

The authors have no conflict of interest to declare.

## PATIENT CONSENT

Written informed consent was obtained from the patient to publish this report in accordance with the journal's patient consent policy.

## References

[1] Toyokawa G , Takenoyama M , Taguchi K , Arakaki K , Inamasu E , Toyozawa R , et al. The first case of lung carcinosarcoma harboring in‐frame deletions at exon19 in the EGFR gene. Lung Cancer. 2013;81(3):491–494. 23891513 10.1016/j.lungcan.2013.06.013

[2] WHO Classification of Tumours Editorial Board . Thoracic Tumours. WHO classification of tumours series. 5th ed. Lyon, France: International Agency for Research on Cancer; 2021.

[3] Bull JC Jr , Grimes OF . Pulmonary carcinosarcoma. Chest. 1974;65(1):9–12. 4809344 10.1378/chest.65.1.9

[4] Lin Y , Yang H , Cai Q , Wang D , Rao H , Lin S , et al. Characteristics and prognostic analysis of 69 patients with pulmonary Sarcomatoid carcinoma. Am J Clin Oncol. 2016;39(3):215–222. 25068469 10.1097/COC.0000000000000101

[5] Koss MN , Hochholzer L , Frommelt RA . Carcinosarcomas of the lung: a clinicopathologic study of 66 patients. Am J Surg Pathol. 1999;23(12):1514–1526. 10584705 10.1097/00000478-199912000-00009

[6] Petrov DB , Vlassov VI , Kalaydjiev GT , Plochev MA , Obretenov ED , Stanoev VI , et al. Primary pulmonary sarcomas and carcinosarcomas – postoperative results and comparative survival analysis. Eur J Cardiothorac Surg. 2003;23(4):461–466. 12694760 10.1016/s1010-7940(03)00024-1

[7] Langer F , Wintzer HO , Werner M , Weber C , Brümmendorf TH , Bokemeyer C . A case of pulmonary carcinosarcoma (squamous cell carcinoma and osteosarcoma) treated with cisplatin and doxorubicin. Anticancer Res. 2006;26(5B):3893–3897. 17094419

[8] Ramalingam SS , Vansteenkiste J , Planchard D , Cho BC , Gray JE , Ohe Y , et al. Overall survival with Osimertinib in untreated, *EGFR*‐mutated advanced NSCLC. N Engl J Med. 2020;382(1):41–50. 31751012 10.1056/NEJMoa1913662

[9] Soria JC , Ohe Y , Vansteenkiste J , Reungwetwattana T , Chewaskulyong B , Lee KH , et al. Osimertinib in untreated EGFR‐mutated advanced non‐small‐cell lung cancer. N Engl J Med. 2018;378(2):113–125. 29151359 10.1056/NEJMoa1713137

[10] Nakagawa K , Garon EB , Seto T , Nishio M , Ponce Aix S , Paz‐Ares L , et al. Ramucirumab plus erlotinib in patients with untreated, EGFR‐mutated, advanced non‐small‐cell lung cancer (RELAY): a randomised, double‐blind, placebo‐controlled, phase 3 trial. Lancet Oncol. 2019;20(12):1655–1669. 31591063 10.1016/S1470-2045(19)30634-5

[11] Zhou Q , Xu CR , Cheng Y , Liu YP , Chen GY , Cui JW , et al. Bevacizumab plus erlotinib in Chinese patients with untreated, EGFR‐mutated, advanced NSCLC (ARTEMIS‐CTONG1509): a multicenter phase 3 study. Cancer Cell. 2021;39(9):1279–1291.e3. 34388377 10.1016/j.ccell.2021.07.005

[12] Watanabe H , Ichihara E , Kayatani H , Makimoto G , Ninomiya K , Nishii K , et al. VEGFR2 blockade augments the effects of tyrosine kinase inhibitors by inhibiting angiogenesis and oncogenic signaling in oncogene‐driven non‐small‐cell lung cancers. Cancer Sci. 2021;112(5):1853–1864. 33410241 10.1111/cas.14801 PMC8088971

[13] Mok TS , Wu Y‐L , Ahn M‐J , Garassino MC , Kim HR , Ramalingam SS , et al. Osimertinib or platinum‐pemetrexed in EGFR T790M‐positive lung cancer. N Engl J Med. 2017;376(7):629–640. 27959700 10.1056/NEJMoa1612674 PMC6762027

[14] Haratake N , Misumi T , Yamanaka T , Seto T . Optimizing sequential treatment with EGFR tyrosine kinase inhibitor with a simulation of the T790M mutation rate in *EGFR*‐mutated lung cancer. JTO Clin Res Rep. 2020;1(4):100085. 34589964 10.1016/j.jtocrr.2020.100085 PMC8474446

